# Whole body kinematic sex differences persist across non-dimensional gait speeds

**DOI:** 10.1371/journal.pone.0237449

**Published:** 2020-08-20

**Authors:** Dustin A. Bruening, Andrew R. Baird, Kelsey J. Weaver, Austin T. Rasmussen

**Affiliations:** 1 Exercise Sciences Department, Brigham Young University, Provo, Utah, United States of America; 2 Mechanical Engineering Department, Brigham Young University, Provo, Utah, United States of America; The Ohio State University, UNITED STATES

## Abstract

Sex differences in human locomotion are of interest in a broad variety of interdisciplinary applications. Although kinematic sex differences have been studied for many years, the underlying reasons behind several noted differences, such as pelvis and torso range of motion, are still not well understood. Walking speed and body size in particular represent confounding influences that hinder our ability to determine causal factors. The purpose of this study was to investigate sex differences in whole body gait kinematics across a range of controlled, non-dimensional walking and running speeds. We hypothesized that as task demand (i.e. gait speed) increased, the influences of modifiable factors would decrease, leading to a kinematic motion pattern convergence between sexes. Motion capture data from forty-eight healthy young adults (24 M, 24 F) wearing controlled footwear was captured at three walking and three running Froude speeds. Spatiotemporal metrics, center of mass displacement, and joint/segment ranges of motion were compared between sexes using 2x6 mixed-model ANOVAs. Three dimensional time-series waveforms were also used to describe the time-varying behavior of select joint angles. When controlling for size, sex differences in spatiotemporal metrics and center of mass displacement disappeared. However, contrary to our hypothesis, sagittal plane ankle, frontal plane pelvis, and transverse plane pelvis and torso range of motion all displayed sex differences that persisted or increased with gait speed. Overall, most spatiotemporal sex differences appear to be related to size and self-selection of gait speeds, while in contrast, sex differences in joint motion may be more inherent and ubiquitous than previously thought. Discussion on potential causal factors is presented.

## 1. Introduction

Sex differences in human walking have been studied for many decades. Much of the early interest was from a social psychology perspective (e.g. [1, 2]), with a primary objective of determining whether sex could be identified through observation of motion-only cues. These studies identified frontal plane torso and pelvis range of motion (RoM) as the most discriminating factors, with males having greater torso RoM and females greater pelvis RoM [2–5]. A number of more empirical biomechanics studies have also been published over the past two decades. These have mostly confirmed frontal plane pelvis differences [6–10], but have been mixed on the torso [6, 11–13]. Across multiple studies, only transverse plane pelvis/hip RoM [6, 7, 14, 15] and sagittal plane ankle RoM [6, 15–21] appear to show additional, consistent sex effects. There is also some limited evidence that transverse plane torso and arm swing may also differ between sexes during walking [6, 22]. The underlying reasons for these sex differences are still not entirely clear, with hypotheses ranging from potentially modifiable socio-cultural influences to evolutionary factors and inherent structural dimorphism.

In addition to walking, sex differences in athletic injury incidence rates (e.g. [23]) have prompted a number of clinical studies looking at sex differences in lower extremity joint mechanics during running. This literature has been more focused, with much of it analyzing lower limb posture during landing, knee and hip loading, or hip muscle activation. Compared with males, females generally appear to land with more knee extension [24] and have greater peak hip adduction and internal rotation [9, 25–28] as well as greater gluteal muscle activation [9, 29]. Most of these differences have been preliminarily attributed to the sexual dimorphism of the human pelvis [30–32].

Despite the substantial body of literature on sex differences in both walking and running kinematics, several major limitations are apparent that hamper our ability to determine causative factors. First, the vast majority of studies have compared sexes at either self-selected walking speeds or fixed running speeds. Gait mechanics are influenced by both body size and speed [33, 34], potentially masking or confounding true sex effects [32]. For example, females often select similar walking speeds to males [35–37] despite having shorter limbs, thus potentially increasing relative task demand. Second, most studies have measured only lower extremity kinematics, typically due to a focus on injury mechanisms or pathologies. Even for lower extremity injuries, the addition of upper body motion may provide additional insights into balance and coordination. Whole body sex effects may also be of interest in applications broader than medicine and sociology, including evolutionary and comparative biology [37–39], human surveillance [40–42], targeted advertising [43], computer animation [44], and even forensics [45].

The overall purpose of the present study was to investigate sex differences in whole body gait kinematics across a range of walking and running speeds. We controlled for the potentially confounding effects of limb length through the use of non-dimensional speeds. We theorized that sex differences consist of two causal categories, which we term inherent (i.e. anatomical structure, physiology, etc.) and modifiable (i.e. sociocultural). We hypothesized that as task demand (i.e. gait speed) increased, the influences of modifiable factors would decrease, leading to a kinematic motion pattern convergence between sexes. We hoped that this approach would help identify true sex differences in gait kinematics and differentiate inherent from modifiable influences.

## 2. Methods

### 2.1 Subjects

Forty-eight young healthy adults (24 M, 24 F) were recruited from the university student body (Table 1). Exclusion criteria consisted of any orthopedic injury or health condition that might affect typical walking or running gait. The study was approved by the Brigham Young University Institutional Review Board (Protocol X17423). All participants were volunteers and signed approved consent forms.

**Table 1:** Sample demographics (mean ± SD).

	Female (n = 24)	Male (n = 24)
Age (yrs)	20.7 ± 2.2	23.0 ± 2.0
Height (cm)	164.8 ± 5.4	180.7 ± 5.8
Mass (kg)	57.3 ± 8.4	82.6 ± 11.0
BMI (kg/m2)	21.1 ± 2.8	25.3 ± 3.1
Leg Length (cm)	85.1 ± 4.0	94.0 ± 3.9

### 2.2 Protocol

Height, weight, and leg length were measured prior to testing. Leg length was measured manually from the right ASIS to the right medial malleolus. A total of sixty-one retro-reflective markers were affixed to each subject according to a custom full-body marker set with a multi-segment foot (see Fig A1 and Table A1 in S1 Appendix). All subjects wore study-specific athletic shoes (Nike T-Lite V) with appropriate cut-outs [46] so that reflective markers could be placed directly on the skin. This shoe is a general cross-training shoe with mid-range stiffness and support. An anterior/posterior split-belt instrumented treadmill (AMTI, inc. Watertown MA USA) was used to control gait speed (note that forces were not used in this study). Participants were allowed as much time as desired to acclimate to the shoes and treadmill prior to data collection. This included walking and running at all of the testing speeds.

After a verbal indication of acclimation (typically a few minutes), each participant was asked to complete a series of three walking speeds followed by three running speeds. To control for differences in height, speeds were non-dimensional, based on each subject’s leg length [47]:

$$F=\frac{v}{\sqrt{gl}}$$

Where *F* = the non-dimensional speed or Froude speed, *v* = speed, *g* = gravity, and *l* = leg length. The tested Froude speeds for walking were 0.32, 0.48, and 0.64, and for running 0.88, 1.12, and 1.36, chosen to represent a wide range of common gait speeds (Table 2).

**Table 2:** Testing speeds.

	Walk			Run		
Froude speed	0.32	0.48	0.64	0.88	1.12	1.36
**M speed (m/s)**	1.0 ± 0.02	1.5 ± 0.03	1.9 ± 0.04	2.7 ± 0.06	3.4 ± 0.07	4.1 ± 0.09
**F speed (m/s)**	0.9 ± 0.02	1.4 ± 0.03	1.8 ± 0.04	2.5 ± 0.06	3.2 ± 0.08	3.9 ± 0.09

The subjects were moved sequentially from the three walking trials to the three running trials, working up from the slowest to the fastest speeds throughout. A speed progression was used to evaluate the effects of increasing speed on sex differences. With each increase in speed, the subjects were given a minimum of 2 minutes to achieve a comfortable, consistent gait prior to collection. Eight seconds of data were then collected at 240 Hz using a 12-camera motion capture system (Vicon, inc. Denver CO, USA).

### 2.3 Data analysis

Data processing was performed in Visual3D software (C-Motion, inc. Germantown MD). A 17-segment full body model was created from a static pose and applied to the walking trials (see Tables A1 and A2 in S1 Appendix for model details). All marker trajectories were filtered using a low pass Butterworth Filter (6 Hz cutoff frequency). Joint angles were then calculated using two different Euler/Cardan angle sequences: One for the pelvis and torso (1-Transverse, 2-Frontal, 3-Sagittal) [48], and one for all other joints (1-Sagittal, 2-Frontal, 3-Transverse).

Joint angle metrics were used for statistical analysis. Metrics consisted of spatiotemporal values (speed, step length, cadence), joint angular displacements (termed range of motion, RoM), and center of mass (CoM) linear displacements, all calculated across the full gait cycle. Spatiotemporal and CoM metrics were evaluated both raw and as non-dimensional quantities, with step length and CoM normalized to leg length and cadence normalized to step length and gravitational acceleration [47]. The specific joints and planes were chosen based on the previously identified sex differences outlined in the introduction (see Table 3 for list of all metrics). Torso and pelvis angles were expressed relative to the laboratory reference frame, while waist represented the angles between the two segments. For ankle and midtarsal joints, the right side was chosen for analysis. Unlike lower extremity kinematics, which are fairly symmetrical, arm swing has previously shown bilateral differences with left RoM greater than right [49]. Thus left shoulder and elbow RoM were included, with bilateral data available in the S1 Appendix. Each participant's metrics were averaged across all the gait cycles associated with the 8-second collection (typically resulting in 5–11 cycles, depending on the subject and speed). For each metric, a 2-way, mixed-model ANOVA was run to determine significant differences between sexes and interactions between sex and speed (SPSS v.25 software). Note that speed main effects were not a focus of this paper, as these have been previously reported (e.g. [34, 50]) and were expected to be significant for all metrics. Partial eta squared values were also reported as a measure of experimental effect strength. To account for multiple comparisons, the Benjamini-Hochberg procedure [51] with a false discovery rate of 0.10 was used to determine statistical significance. This procedure controls the false discovery rate by ranking the p-values and comparing them to a critical value. The procedure was run twice, once for sex main effects and again for interaction main effects.

**Table 3:** ANOVA main effects *p*-values for spatiotemporal metrics, center of mass (CoM) displacements, and range of motion (RoM) metrics (α = 0.05). The Benjamini-Hochberg technique was used to control the false discovery rate, with * indicating significance. Partial eta squared values are also presented as a measure of effect strength.

	ANOVA main effects *p*-values		Effect Size (Partial η^2^)	
	Sex	Interaction	Sex	Interaction
***Spatiotemporal***				
Speed	<0.001*	<0 .001*	0.562	0.562
Step Length	<0.001*	<0.001*	0.311	0.119
Step Length (ND)	0.124	0.489	0.051	0.019
Cadence	0.047	0.149	0.083	0.035
Cadence (ND)	0.106	0.359	0.056	0.023
***ND CoM displacement***				
CoM (Vertical)	0.592	0.042	0.006	0.048
CoM (M/L)	0.887	0.007*	0.001	0.037
***Range of motion***				
Ankle (Sagittal)	<0.001*	0.008*	0.221	0.066
Midtarsal (Sagittal)	0.734	0.333	0.003	0.024
Pelvis (Frontal)	<0.001*	0.092	0.441	0.040
Pelvis (Transverse)	<0.001*	0.006*	0.316	0.067
Torso (Frontal)	0.492	0.117	0.010	0.037
Torso (Transverse)	<0.001*	<0.001*	0.571	0.620
Waist (Frontal)	0.002*	0.550	0.183	0.017
Waist (Transverse)	<0.001*	<0.001*	0.428	0.425
LShoulder (Sagittal)	0.003*	0.042	0.182	0.049
LElbow (Sagittal)	0.687	<0.001*	0.116	0.004

A few of the variables were also displayed as waveforms to show differences across time (only descriptively). These consisted of the sagittal plane ankle and frontal/transverse plane pelvis and torso angles. These angle trajectories were broken into gait cycles and time-normalized to percent gait cycle. For each subject, a mean waveform was created by averaging across trials, then these mean waveforms were averaged across subjects and presented as three dimensional graphs (time, angle, speed).

## 3. Results

### 3.1 Spatiotemporal metrics and center of mass (Fig 1, Tables 3 and A3 in S1 Appendix)

**Fig 1:**
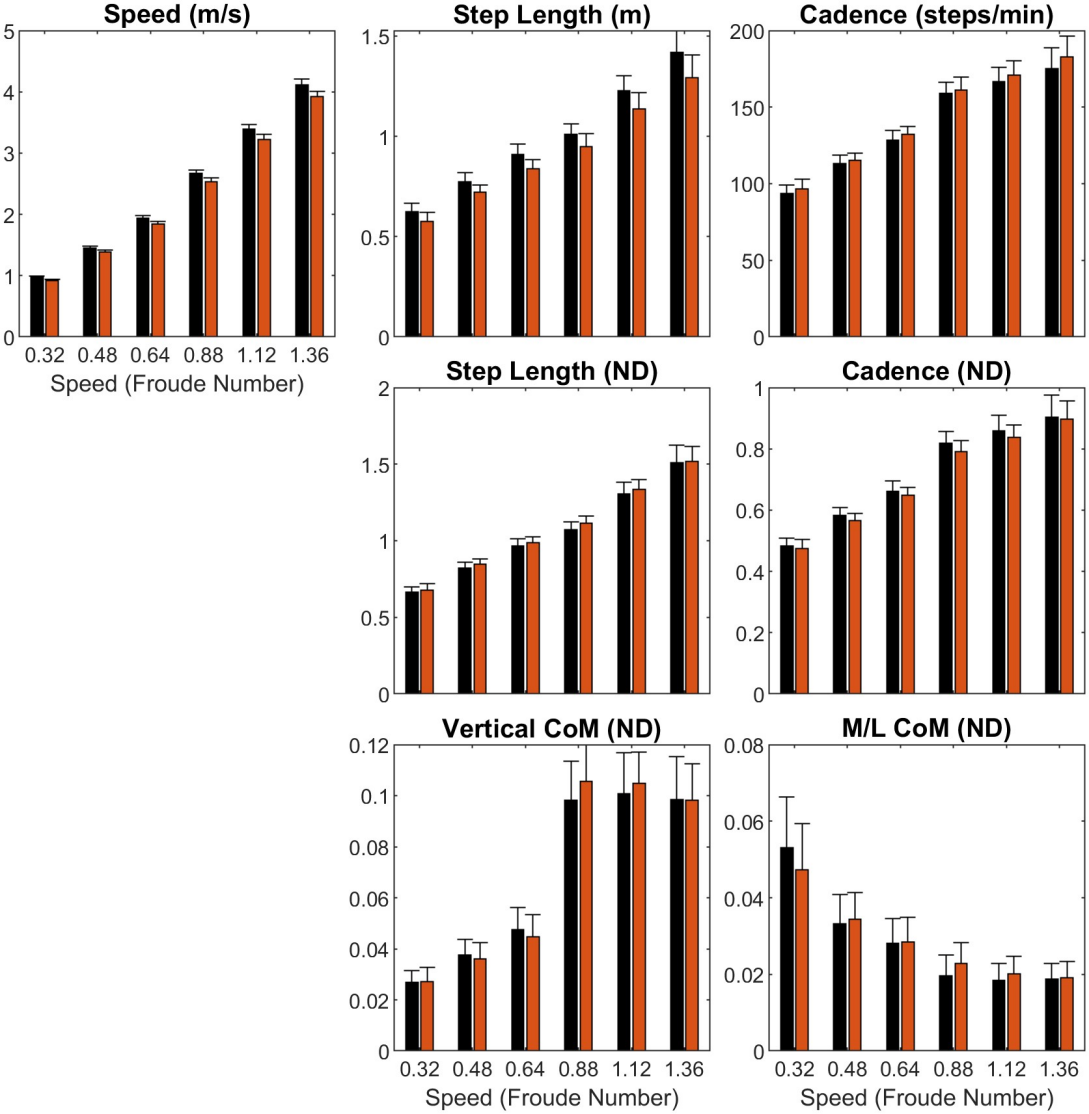
Spatiotemporal and center of mass (CoM) metrics. Clustered bar charts showing sex differences across speeds (means ± SD). Legend: Black = male, red = female.

Across speeds, females had shorter step lengths (*p*<0.001) than males, with step length differences increasing with speed (*p*<0.001). However, this sex difference disappeared when the step length was non-dimensionalized. No sex differences were seen in non-dimensional vertical and M/L center of mass displacements; however, there was a sex-speed interaction effect in M/L CoM (*p* = 0.007).

### 3.2 Joint angle RoMs (Figs 2 and 3, Tables 3 and A3 in S1 Appendix)

**Fig 2:**
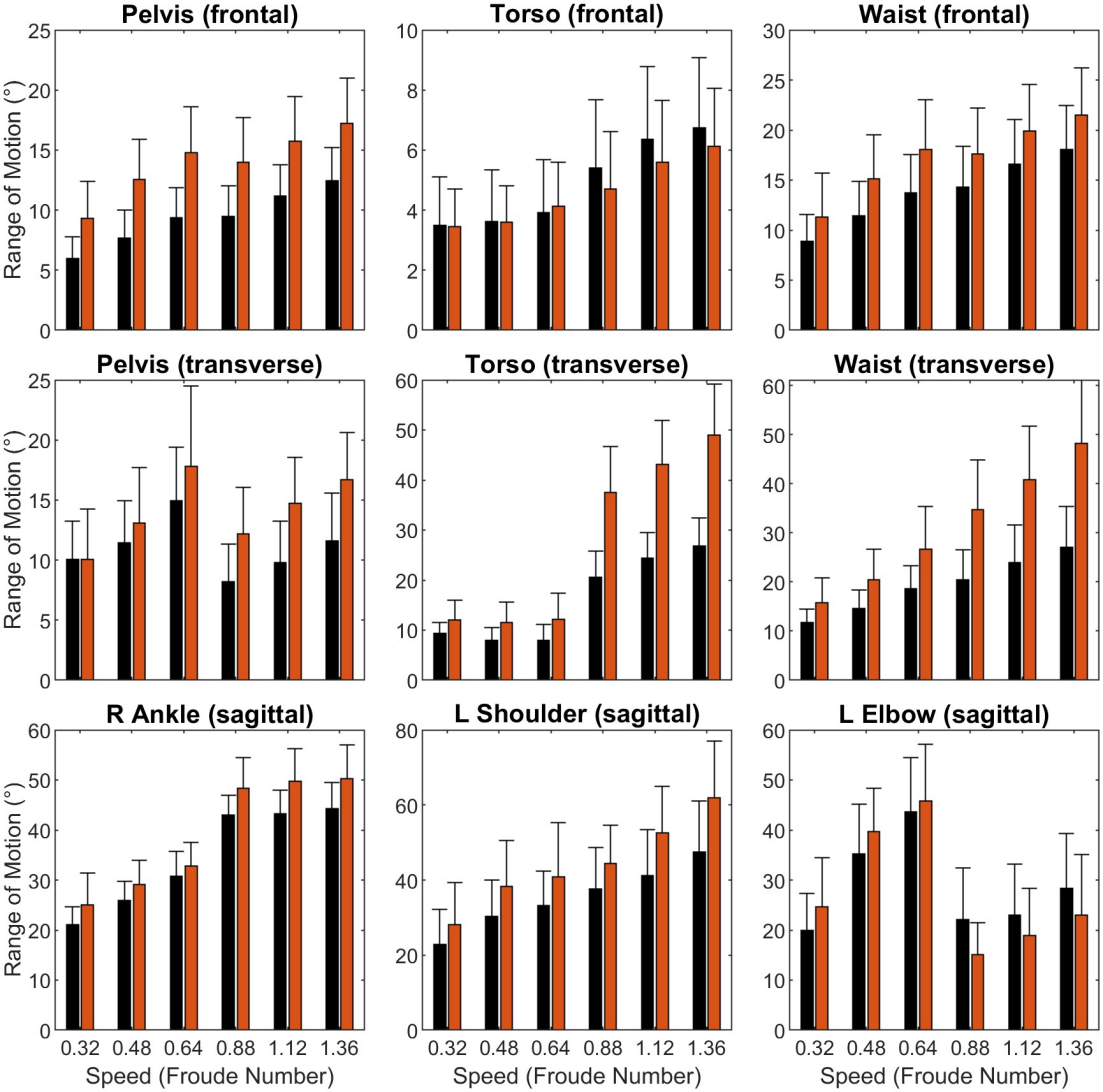
Range of motion (RoM) metrics. Clustered bar charts showing sex differences across speeds (means ± SD). Legend: Black = male, red = female.

**Fig 3:**
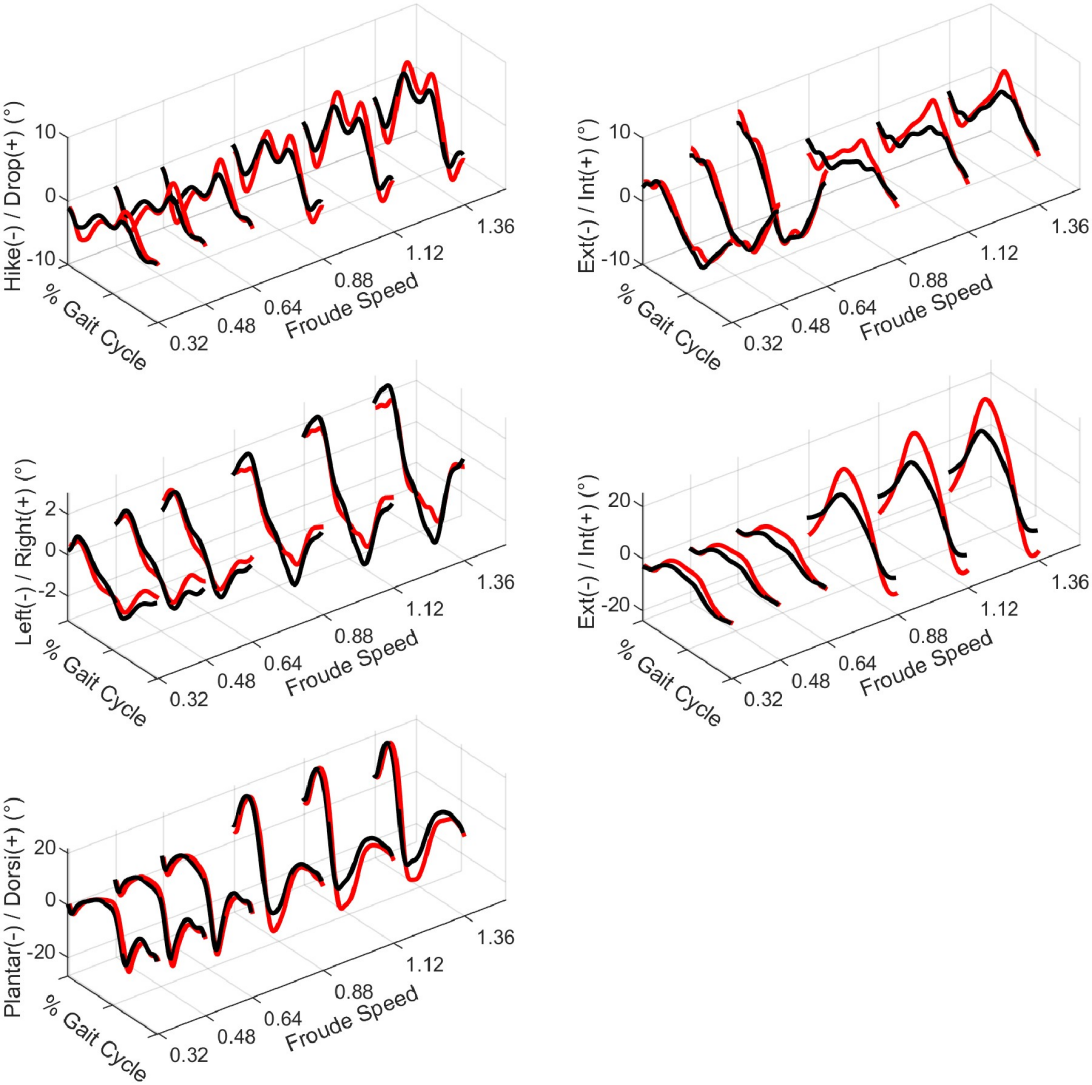
Time-series waveforms for A) frontal plane pelvis, B) transverse pelvis, C) frontal torso, D) transverse torso, and E) sagittal ankle. Three-dimensional graphs show mean waveforms time normalized to % gait cycle, displayed across speeds.

Total sagittal plane ankle RoM was greater in females across speeds (*p*<0.001), with slightly greater differences in running than walking (*p* = 0.008). These differences arose primarily from increased female plantarflexion in pre-swing and initial swing, and are particularly prominent in the running trials (Fig 3). No sex differences were found in midtarsal RoM.

Frontal plane pelvis RoM was much greater in females (*p*<0.001), with visually more prominent peaks and potentially sharper changes between peaks (e.g. increased angular velocity, Fig 3). No statistical sex difference was found in frontal plane torso RoM, leaving the waist with sex differences similar to the pelvis (*p* = 0.002). No sex-speed interaction effects were present in any of these three frontal plane metrics. In the transverse plane, female pelvis, waist, and torso RoMs were all significantly greater than male RoM (*p*<0.001). Differences again visually appeared to consist of greater peaks and angular velocities (Fig 3). All three metrics also showed significant sex-speed interactions (p<0.006), with sex differences visually much larger in running than walking (Fig 2). At the pelvis, there was a large drop in RoM during the walk to run transition, while at the torso there was a large jump in RoM during the same transition. This resulted in a fairly linear increase in waist RoM.

Left shoulder RoM was greater in females (*p* = 0.003). Left elbow RoM was not different between sexes, but did show a significant interaction (*p*<0.001), appearing slightly higher during walking but lower during running. Elbow RoM was also much lower in running than in walking.

## 4. Discussion

The purpose of this study was to investigate sex differences in gait kinematics across non-dimensional speeds. We created a general hypothesis that the influences of modifiable factors would decrease as speed increased. Our results, however, showed that most sex differences persisted or even increased with speed. We believe that this suggests that inherent factors predominantly drive these differences. Discussion below is organized around metrics and body regions.

### 4.1 Spatiotemporal metrics

The vast majority of research on sex differences in gait have employed self-selected walking speeds. This is justified in a broad context, as terrestrial animals typically choose preferred speeds that minimize energy cost [52]. However, external factors also influence speed [36]. For example, females often select walking speeds similar to males [35, 37] despite having shorter limbs, perhaps for sociocultural reasons [37, 53]. We chose to use fixed Froude speeds in an attempt to reduce external influences on speed, minimize the influence of size, and place males and females on roughly comparable gait efficiency terms [54]. Even at controlled speeds, step length and cadence are still partially dependent on limb length [47]. When these step lengths and cadences were also normalized, we showed no differences between sexes in either metric. This is in contrast to typical dimensional analyses that show longer step lengths in males and higher cadences in females (e.g. [35, 36]). The present results suggest that these differences likely primarily represent differences in size and self-selection of gait speeds rather than inherent sex differences.

One attractive explanation for findings of increased pelvic obliquity and rotation and increased ankle plantarflexion in females is in the control of step length. These three variables can theoretically influence step length by making the limb’s effective length longer at initial contact and toe-off [55] and it is tempting to attribute associated sex differences, at least in part, to an attempt by females to increase walking speed relative to stature [20, 38, 56]. Our findings, however, show that these sex effects are present even when size and speed are controlled for. To further confirm this finding, we performed (post-hoc) a stepwise multiple linear regression, predicting step length from leg length, pelvic obliquity, pelvic rotation, ankle plantarflexion, sex, and the interactions of the former variables with sex (SPSS v.25 software). We found a significant correlation with leg length (p<0.011, r = 0.36), but none of the other variables were significantly correlated and used by the model (α = 0.05). While we cannot conclude that these mechanisms are not used to modulate step length in other contexts [57], our results suggest that at equal Froude speeds, step length is not related to these variables, and that sex differences exist independent from step length. These findings are in line with previous studies showing minimal pelvic motion influences on step length [58, 59] and similar coordination scaling with speed between sexes [60].

### 4.2 CoM

A fair amount of literature has been devoted to sex differences in locomotor efficiency, often associating this with pelvis motion and CoM displacement. Traditionally, the wider female pelvis [32] has been thought to represent a functional compromise between childbirth and walking efficiency, the latter thought to be lower in females due to decreased hip abductor lever arms that require greater muscle forces [39]. Several studies have challenged this assertion, however, suggesting that walking efficiency may be equal [37, 39] or even greater in females [37, 56], perhaps due to a smoother vertical CoM path [10, 38, 56]. We did not evaluate walking efficiency in this study, but our CoM and pelvis motion results do provide some insight into this debate. First, we saw large pelvis motion sex differences, but we did not see a consistent sex difference in either vertical or horizontal CoM displacements. We did note a significant speed-sex interaction effect (e.g. slightly greater male M/L CoM displacment in walking, reverse in running). This may suggest that while CoM displacement is highly dependent on speed, with vertical displacement increasing with speed and horizontal displacement decreasing [61], the manner in which it changes with speed may differ subtly between sexes. This interaction may merit additional investigation. Second, our results suggest that previous reports of CoM sex differences [10, 62] are likely confounded by size or speed effects, or are speed dependent, and should be evaluated in those specific contexts. Overall, CoM displacement likely has only a minimal connection with pelvis motion [63] and, in line with several other previous studies [64, 65], may provide little insight into locomotor efficiency.

### 4.3 Pelvis

While sex differences in pelvis motion have been studied extensively, the underlying reasons for these differences are still unclear. Greater frontal plane RoM has consistently been noted in females compared to males across studies [6–10], while transverse plane RoM differences have been less consistent [6, 7, 14, 15]. Our results show sex differences in both planes that persisted across speeds. However, transverse plane differences were small during slow walking and increased across speeds, which may be one reason for previous inconsistent results. Two theories on increased female pelvis motion have already been addressed above—modulating step length and decreasing or smoothing vertical CoM displacement. Our results suggest that neither of these theories are likely primary factors. A number of studies have also suggested that the greater pelvic obliquity in females is in part due to sociocultural factors [4, 66, 67]. We cannot completely rule this out, however, the persistence or increase of pelvis motion differences with task demand suggests that most sex differences are at least not consciously modified [66]. Structurally, the most apparent sex difference is the wider, broader female pelvis and wider pelvic aperture [32]. This contributes to a greater Q-angle in females and greater hip adduction when weight bearing (e.g. [9, 25]), along with potential sex differences in muscle activation [9, 29, 68]. While these differences likely contribute to sex differences in knee injury incidence rates [23], they do not yet fully explain sex differences in pelvis motion. Further exploration is needed to identify specific causal factors.

### 4.4 Upper body

Despite social science research identifying frontal plane torso motion as a prominent sex-distinguishing feature [1–3, 5, 67], empirical studies on this are mixed. Several accelerometry based studies [69–72] have shown lower M/L and A/P linear torso and head accelerations in females along with greater attenuation of accelerations from pelvis to head. Bruening et al. [6] also found lower frontal plane RoM in females along with similar acceleration attenuation; however, other studies have not [11–13]. We initially thought that this motion in particular might have substantial sociocultural influences [4, 5, 67, 73] and would therefore decrease as task demand increased, particularly during high running speeds when efficiency is most likely to drive movement. Yet, this metric increased across speeds with no significant sex difference main effects. In addition, means appeared to slightly diverge during running, and it is possible that sex differences might be evident with additional subjects or a focus only on running. When taken across all studies, we suggest that frontal plane torso sex differences may exist but are likely subtle, should increase with speed, and thus should contain inherent factors.

In our study, we saw substantial sex effects in transverse plane torso motion, particularly during running, with female RoM nearly double that of males at the fastest running speed. Much of this may be passively driven by the greater female pelvis rotation and higher raw cadence values. However, there were also some contrasts between the pelvis and torso across speeds. During walking, pelvis RoM was greater than torso RoM, while during running, pelvis RoM decreased slightly and torso RoM increased markedly. Interestingly, the transverse plane waist angles increased in a consistent, predictable manner across all speeds, with linearly greater divergence between sexes as speed increases. This consistency in waist motion between sexes was noted previously [6] and may have significance in some form of modulation of pelvis and torso rotations. The increased waist angles in females may also have some influence on spine torque and the higher incidence of low back pain seen in females [74].

The greater female torso rotation is likely the primary driver behind the additional shoulder RoM seen in females, as much of arm swing is passive [75, 76]. However, shoulder RoM did not perfectly mirror torso rotation, suggesting that there may be some additional sex differences in structure and/or the active portion of arm control. Additionally, while shoulder motion was elevated in females, elbow motion was not, and may actually be greater in males when running. In this study we focused primarily on left arm motion as it has been shown to be greater than right arm motion during walking, regardless of handedness [49]. While we did not analyze arm symmetry, our results (Table A3 in S1 Appendix, S1 Appendix) support this assertion during walking, but suggest that this may not be true during running. Arm motion has traditionally received little attention in the literature, but may be of interest in numerous identification based applications, as it is possible that sex effects could be more noticeable in arm swing than in torso motion (or using a combination of the two). These applications range from medical (e.g. Parkinson’s disease [77]) to surveillance [40–42], targeted advertising [43], computer animation [44], and forensics [45].

### 4.5 Foot and ankle

Despite significant sex differences in ankle RoM across numerous studies [6, 15–19], very little discussion has been devoted to this topic. This is likely due to the relatively small effect sizes, resulting in the ankle being a side note to more clinically prominent pelvis and hip differences. Although small, the differences are remarkably consistent across studies. Recently, two studies [20, 21] employed multi-segment foot models to look at the foot in more detail. Using self-selected walking speeds, Lee et al. [20] showed that the greater female RoM was confined to the ankle and not the midfoot, while Takabayashi et al.[21] showed similar results at slow running speeds. Our results are consistent with both of these studies, confirming that this is not an artifact of gait speed. We also add that the bulk of this RoM difference is from increased peak female plantarflexion during push off, and that the differences are much greater in running than in walking. Several potential explanatory factors have been proposed. Lee et al.[20] suggested increased generalized joint laxity or a compensation for shorter leg lengths to achieve faster walking speeds. Our results suggest that neither of these are likely. Leg length was mostly accounted for through non-dimensional speeds, while laxity should be more apparent in the midfoot or during dorsiflexion, as opposed to ankle plantarflexion at push off. Although our use of footwear could have affected foot laxity and we can’t completely rule this theory out, there appears to be good agreement among these studies despite differing footwear conditions. Takabayahi et al.[21] also proposed weaker female ankle musculature and potential muscle insertion differences [78] as possible factors. However, gastrosoleus muscle activity is typically waning during pre-swing, and much of the final plantarflexion is theorized to arise from elastic energy storage and return [79, 80]. We previously postulated [6] that the proportionally shorter female foot length [40, 81] might influence plantarflexion, for instance by requiring a greater plantarflexion push off angle to reach the same vertical height change as a longer foot (see also [63, 82, 83]). Finally, in closed-chain motion, proximal pelvis and distal ankle influences may be coupled. Future studies exploring structural and muscular sex differences may help determine these causal factors.

### 4.6 Limitations

While we sought to fill gaps in the literature on this topic, designing a study to isolate sex differences in human movement inherently involves a number of tradeoffs with associated limitations. We focused on a narrow, college-age student demographic. This provided a homogeneous study sample for robust statistical treatment; however, it may not fully generalize to other demographics, particularly aging [36, 84] or pathological populations that are often of interest in clinical applications. Related, there were some demographics differences between groups in age and BMI. These differences are representative of this population, but should be noted as they could influence the analysis. Another tradeoff was in the use of standardized footwear. This was chosen to remove footwear variance as a potential confounder and to allow for markers to be placed on the foot through cut-outs in the shoe. It is possible that standardized shoes may introduce some variance associated with shoe comfort, but the alternative—variance associated with shoe materials, style, and function—was deemed worse when analyzing sex effects. Participants were also given plenty of time to acclimate to the shoes prior to testing. We also conducted our studies on a treadmill rather than overground. This was a decided logistical advantage for acclimation, speed and external factors control, and ease of data collection. While some studies have found subtle differences between treadmill and overground walking, these are primarily confined to gait variability and rehabilitation outcomes [85], and should have minimal effect on mean joint RoM [86]. We also made the decision a priori to follow a speed progression, rather than randomize the order of testing speeds. We felt that the speed progression best represented increasing demand, i.e. our original hypothesis revolved around the question of what happens when participants are tasked more. While we do not anticipate order effects due to learning or fatigue, we acknowledge the possibility of unforeseen effects due to testing order. Finally, the use of fixed, non-dimensional speeds was chosen to address the confounding influence of size. While this is a common method of controlling for size differences, one limitation is that absolute changes in speed differ slightly for each participant. In addition, our Froude speeds may differ slightly from other publications due to a lack of standard terminology. For example, the Froude speed has sometimes been defined as v^2^/gl [87] instead of v/sqrt(gl) [47], while leg length measurement protocols also differ among studies. This lack of standardized accounting resulted in a walk to run transition that did not occur at the theoretical Froude speed of 0.5 [87], but, more importantly, the evaluated speeds were chosen to represent a wide range of common locomotor speeds [35].

## 5. Conclusions

In general, our main hypothesis was not upheld, as sex differences in prominent joint RoMs persisted or increased with speed. However, our results helped clarify the influence of body size and speed on locomotor kinematic sex differences and provided some insight into many of the current debates over potential causal factors. These results suggest that sex differences may be more inherent and ubiquitous than previously thought. Still, many of the reasons behind these sex differences are still unclear and require further investigation. Given the numerous interdisciplinary interest in human locomotion, we hope that these results will assist in the design of targeted studies on this topic. A number of applications were presented throughout this paper. Additional interest has been noted in osteoarthritis [88, 89], patellofemoral pain [90], military training [91, 92], shoe design [93], and multiple sclerosis [94], to name just a few.

## Supporting information

S1 Appendix (PDF)
