# Supplementary material for: Whole body kinematic sex differences persist across non-dimensional gait speeds
Source: PLoS One. 2020 Aug 20;15(8):e0237449. doi: 10.1371/journal.pone.0237449 (PMC7440644; doi:10.1371/journal.pone.0237449)
Supplement: S1 Appendix — (PDF) [file pone.0237449.s001.pdf]

APPENDIX:

Model: A custom 61-marker, 19-segment model was employed. Marker positions are shown in Figure A1, with placement descriptions in Table A1. Model segment definitions are shown in Table A2.

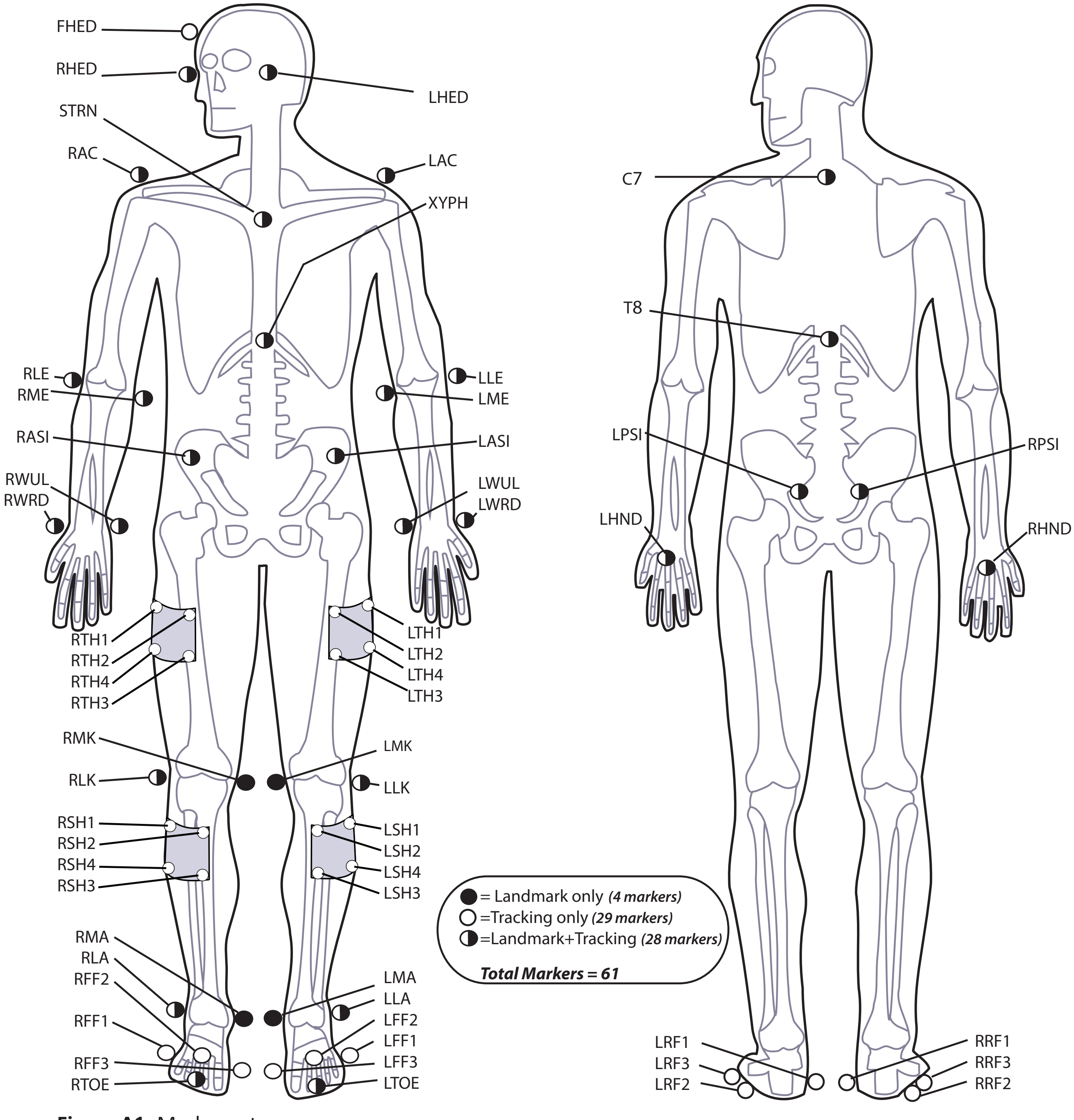

Figure A1: Marker set.

Table A1: Markers and landmarks. Only the right side is listed for bilateral markers.

NAME	PLACEMENT	NAME	PLACEMENT
FHED	Forehead (arbitrary)	RASI	Anterior superior iliac spine
RHED	Just anterior to the tragus	RPSI	Posterior superior iliac spine
C7	Cervical spine 7 spinous process	RTH1-4	4-Marker thigh cluster
STRN	Sternum, just distal to jugular notch	RLK	Lateral femoral epicondyle
XYPH	Xyphoid process	RMK	Medial femoral epicondyle
T8	Thoracic 8 spinous process	RSH1-4	4-Marker shanke cluster
RAC	Acromioclavicular joint	RLA	Lateral malleolus
RLE	Lateral humeral epicondyle	RMA	Medial malleolus
RME	Medial humeral epicondyle	RRF1-3	Calcaneus (rearfoot), 3-markers
RWUL	Just proximal to ulnar styloid process	RFF1-3	Metatarsals (forefoot), 3-markers
RWRD	Just proximal to radial styloid process	RTOE	Between 2nd and 3rd phalanges
RHND	Metacarpal head 3		
**Virtual Landmarks**			
HDC	Midpoint between RHED and LHED		
NKC	Midpoint between C7 and STRN		
TRC	Midpoint between XYPH and T8		
RSJ	RAC projected downward (12% RAC-LAC) and lateral (5% RAC-LAC).*		
REJ	Midpoint between RLE and RME		
RWJ	Midpoint between RWUL and RWRD		
MASI	Midpoint between RASI and LASI		
MPSI	Midpoint between RPSI and LPSI		
RHJ	Offsets according to Harrington et al.**		
RKJ	Midpoint between RLK and RMK		
RAJ	Midpoint between RLA and RMA		
RAJp	RAJ projected downward to height of RTOE		

* Modified from Rab et al. [1].

** Harrington et al. [2].

Table A2: Model segment definitions.

Segment	Long Axis	Plane	Tracking
Head	NKC to HDC	NKC, RHED, LHED (frontal)	FHED, RHED, LHED
Torso	TRC to NKC	C7, STRN, XYPH, T8 (sagittal)	C7, STRN, XYPH, T8
R. Clavicle	NKC to RSJ	NKC, RSJ, STRN (transverse)	NKC, RAC, STRN
R. Upper Arm	RSJ to REC	RSJ, RLE, RME (frontal)	R. Clavicle end, RLE, RME
R. Forearm	REC to RWC	RLE, RME, RWC (frontal)	RLE, RME, RWC
R. Hand	RWC to RHND	RWUL, RWRD, RHND (frontal)	RWUL, RWRD, RHND
Pelvis	MASI to PASI	RASI, LASI, RPSI, LPSI (frontal)	RASI, LASI, RPSI, LPSI
R. Thigh	RHJ to RKJ	RHJ, RLK, RMK (frontal	RTH1-4
R. Shank	RKJ to RAJ	RKJ, RLA, RMA (frontal)	RSH1-4
R. Rearfoot	RAJp to RTOE	RAJ, RAJp, RTOE (sagittal)	RRF1-3
R. Forefoot	RAJp to RTOE	RAJ, RAJp, RTOE (sagittal)	RFF1-3

Table A3: Metric magnitudes. Mean ± SD for all metrics included in the study (ND = non-dimensional).

		**Walk**			**Run**		
**Froude speed (ND)**		**0.32**	**0.48**	**0.64**	**0.88**	**1.12**	**1.36**
**Speed (m/s)**	M	1.0 ± 0.02	1.5 ± 0.03	1.9 ± 0.04	2.7 ± 0.06	3.4 ± 0.07	4.1 ± 0.09
	F	0.9 ± 0.02	1.4 ± 0.03	1.8 ± 0.04	2.5 ± 0.06	3.2 ± 0.08	3.9 ± 0.09
**Step Length (m)**	M	0.62 ± 0.04	0.77 ± 0.05	0.91 ± 0.05	1.01 ± 0.05	1.23 ± 0.08	1.42 ± 0.11
	F	0.57 ± 0.04	0.72 ± 0.04	0.84 ± 0.04	0.95 ± 0.07	1.14 ± 0.08	1.29 ± 0.11
**Step Length (ND)**	M	0.66 ± 0.04	0.82 ± 0.04	0.97 ± 0.05	1.07 ± 0.05	1.31 ± 0.08	1.51 ± 0.11
	F	0.68 ± 0.04	0.85 ± 0.03	0.99 ± 0.04	1.11 ± 0.05	1.34 ± 0.06	1.52 ± 0.10
**Cadence (steps/min)**	M	93.6 ± 5.5	113.0 ± 5.6	128.3 ± 6.7	158.9 ± 7.2	166.5 ± 9.6	175.0 ± 13.8
	F	96.7 ± 6.4	115.4 ± 4.5	132.2 ± 5.3	161.1 ± 8.4	170.9 ± 9.4	182.9 ± 13.6
**Cadence (ND)**	M	0.483 ± 0.027	0.582 ± 0.026	0.661 ± 0.034	0.819 ± 0.038	0.859 ± 0.052	0.903 ± 0.073
	F	0.474 ± 0.031	0.566 ± 0.022	0.649 ± 0.026	0.790 ± 0.036	0.838 ± 0.039	0.897 ± 0.059
**CoM-Vertical (ND)**	M	0.027 ± 0.005	0.038 ± 0.006	0.047 ± 0.009	0.098 ± 0.015	0.101 ± 0.016	0.098 ± 0.017
	F	0.027 ± 0.006	0.036 ± 0.006	0.045 ± 0.009	0.106 ± 0.014	0.105 ± 0.012	0.098 ± 0.014
**CoM-M/L (ND)**	M	0.053 ± 0.013	0.033 ± 0.008	0.028 ± 0.006	0.020 ± 0.005	0.018 ± 0.005	0.019 ± 0.004
	F	0.047 ± 0.012	0.034 ± 0.007	0.028 ± 0.006	0.023 ± 0.005	0.020 ± 0.005	0.019 ± 0.004
**Ankle-Sagittal (°)**	M	21.1 ± 3.6	26.0 ± 3.8	31.0 ± 4.6	43.2 ± 3.9	43.4 ± 4.7	44.4 ± 5.2
	F	25.0 ± 6.4	29.1 ± 4.9	32.8 ± 4.7	48.4 ± 6.0	49.7 ± 6.6	50.2 ± 6.8
**Midtarsal-Sagittal (°)**	M	10.9 ± 2.7	12.9 ± 4.2	13.5 ± 3.7	13.8 ± 4.7	17.6 ± 5.8	22.2 ± 8.7
	F	11.8 ± 2.3	12.3 ± 2.8	14.0 ± 4.1	13.9 ± 5.4	16.9 ± 7.7	19.7 ± 6.0
**Pelvis-Frontal (°)**	M	6.0 ± 1.8	7.6 ± 2.3	9.4 ± 2.5	9.5 ± 2.5	11.2 ± 2.6	12.5 ± 2.7
	F	9.3 ± 3.1	12.6 ± 3.4	14.8 ± 3.8	14.0 ± 3.7	15.7 ± 3.7	17.2 ± 3.8
**Pelvis-Transverse (°)**	M	10.0 ± 3.2	11.4 ± 3.5	14.9 ± 4.5	8.2 ± 3.2	9.8 ± 3.5	11.6 ± 3.9
	F	10.0 ± 4.2	13.1 ± 4.6	17.8 ± 6.7	12.2 ± 3.8	14.7 ± 3.8	16.7 ± 3.9
**Torso-Frontal (°)**	M	3.5 ± 1.6	3.6 ± 1.7	3.9 ± 1.8	5.4 ± 2.3	6.4 ± 2.4	6.7 ± 2.3
	F	3.5 ± 1.2	3.6 ± 1.2	4.1 ± 1.5	4.7 ± 1.9	5.6 ± 2.1	6.1 ± 1.9
**Torso-Transverse (°)**	M	9.4 ± 2.1	8.0 ± 2.6	7.9 ± 3.2	20.6 ± 5.1	24.4 ± 5.1	26.8 ± 5.6
	F	12.0 ± 3.9	11.5 ± 4.0	12.2 ± 5.2	37.5 ± 9.2	43.1 ± 8.9	49.0 ± 10.1
**RShoulder-Sagittal (°)**	M	20.9 ± 9.2	26.9 ± 10.3	33.5 ± 11.0	37.4 ± 8.8	42.1 ± 10.6	47.8 ± 11.9
	F	23.2 ± 11.5	34.0 ± 14.4	37.9 ± 16.0	43.4 ± 9.3	50.8 ± 11.7	60.6 ± 14.1
**LShoulder-Sagittal (°)**	M	22.8 ± 9.4	30.3 ± 9.7	33.2 ± 9.2	37.6 ± 11.2	41.2 ± 12.2	47.4 ± 13.7
	F	28.1 ± 11.2	38.3 ± 12.3	40.8 ± 14.5	44.5 ± 10.2	52.5 ± 12.4	61.9 ± 15.1
**RElbow-Sagittal (°)**	M	17.1 ± 5.6	30.1 ± 7.8	40.4 ± 12.8	26.5 ± 11.3	28.0 ± 11.1	34.9 ± 12.7
	F	21.2 ± 9.3	37.1 ± 14.4	42.0 ± 13.5	17.0 ± 7.4	20.2 ± 9.8	24.5 ± 11.9
**LElbow-Sagittal (°)**	M	19.9 ± 7.4	35.2 ± 9.9	43.6 ± 10.8	22.1 ± 10.3	23.0 ± 10.2	28.4 ± 10.9
	F	24.7 ± 9.8	39.7 ± 8.7	45.7 ± 11.4	15.1 ± 6.4	18.9 ± 9.5	23.0 ± 12.1
**Waist-Frontal (°)**	M	8.9 ± 2.7	11.4 ± 3.5	13.7 ± 3.8	14.3 ± 4.0	16.6 ± 4.5	18.1 ± 4.4
	F	11.3 ± 4.4	15.1 ± 4.4	18.1 ± 4.9	17.6 ± 4.6	19.9 ± 4.6	21.5 ± 4.7
**Waist-Transverse (°)**	M	11.8 ± 2.8	14.6 ± 3.8	18.6 ± 4.7	20.4 ± 6.1	23.9 ± 7.7	27.1 ± 8.3
	F	15.8 ± 5.0	20.5 ± 6.2	26.7 ± 8.6	34.7 ± 10.2	40.8 ± 10.9	48.2 ± 12.9

Appendix References:

1. Rab G, Petuskey K, Bagley A (2002) A method for determination of upper extremity kinematics. Gait & posture 15: 113-119.
2. Harrington M, Zavatsky A, Lawson S, Yuan Z, Theologis T (2007) Prediction of the hip joint centre in adults, children, and patients with cerebral palsy based on magnetic resonance imaging. Journal of biomechanics 40: 595-602.
